# Supplementary material for: Methods matter: the relationship between strength and hypertrophy depends on methods of measurement and analysis
Source: PeerJ. 2018 Jun 27;6:e5071. doi: 10.7717/peerj.5071 (PMC6026459; doi:10.7717/peerj.5071)
Supplement: Supplemental Information 2 [file peerj-06-5071-s003.pdf]

Appendix A

Vigotsky *et al.*

Here, we show that the partial η^2 calculated using type III sums of squares (SS) is equivalent to how the variance accounted for (VAF) is derived for our hierarchical linear model (HLM). Notation is as follows: SSR = SS due to the regression; SSE = SS due to error (residual); SST = SS total $(SSR + SSE)$; s^2 = explained absolute sample variance; s^2_{residual} = unexplained absolute sample variance.

Proof. Partial η^2 is equivalent to HLM VAF.

$$1 - \frac{s^2}{s^2_{\text{uncond}}} = \frac{SS_{size}}{SS_{size} + SS_{\text{residual}}} \tag{1}$$

$$= \frac{SSR\left\{\text{size} \mid \text{subject}\right\}}{SSR\left\{\text{size} \mid \text{subject}\right\} + SSE\left\{\text{size} \mid \text{subject}\right\}} \tag{2}$$

$$= \frac{\frac{1}{n-1}SSR\left\{\text{size} \mid \text{subject}\right\}}{\frac{1}{n-1}SSR\left\{\text{size} \mid \text{subject}\right\} + \frac{1}{n-1}SSE\left\{\text{size} \mid \text{subject}\right\}} \tag{3}$$

$$= \frac{s^2\left\{\text{size} \mid \text{subject}\right\}}{s^2\left\{\text{size} \mid \text{subject}\right\} + s^2_{\text{residual}}\left\{\text{size} \mid \text{subject}\right\}} \tag{4}$$

Following the $SST = SSR + SSE$ relationship, we know that $s^2_{\text{residual}}\left\{\text{subject}\right\} = s^2\left\{\text{size} \mid \text{subject}\right\} + s^2_{\text{residual}}\left\{\text{size} \mid \text{subject}\right\}$, so

$$= \frac{s^2\left\{\text{size} \mid \text{subject}\right\}}{s^2_{\text{residual}}\left\{\text{subject}\right\}} \tag{5}$$

$$= \frac{s^2_{\text{residual}}\left\{\text{subject}\right\} - s^2_{\text{residual}}\left\{\text{size} \mid \text{subject}\right\}}{s^2_{\text{residual}}\left\{\text{subject}\right\}} \tag{6}$$

$$= 1 - \frac{s^2_{\text{residual}}\left\{\text{size} \mid \text{subject}\right\}}{s^2_{\text{residual}}\left\{\text{subject}\right\}} \tag{7}$$

Finally, the sample variance of the residuals from the HLM were defined such that $s^2 = s^2_{\text{residual}}\left\{\text{size} \mid \text{subject}\right\}$ and $s^2_{\text{uncond}} = s^2_{\text{residual}}\left\{\text{subject}\right\}$; therefore,

$$= 1 - \frac{s^2}{s^2_{\text{uncond}}} \qquad \square$$
